# 536 Early Post-operative Dressing Removal Reduced Number of Antibiotic Days

**DOI:** 10.1093/jbcr/iraf019.165

**Published:** 2025-04-01

**Authors:** Brittany Blass, Scott Mueller, Justin Burleson, Bethany Schmoker, Arek Wiktor, Cameron Gibson

**Affiliations:** Burn and Frostbite Center at University of Colorado Health; University of Colorado Health; University of Colorado Health; University of Colorado Health; University of Colorado Health; University of Colorado Health

## Abstract

**Introduction:**

Burn patients are vulnerable to infections, often requiring antibiotics for treatment. Prolonged antibiotic use increases the risk of multidrug-resistant organisms, secondary infections like Clostridioides difficile, and adverse drug reactions while disrupting the microbiome and delaying healing. Identifying ways to reduce number of antibiotic days in burn patients is essential. This project aimed to reduce the number of antibiotic days by changing the post-operative dressing takedown (POTD) from day 5 to day 3.

**Methods:**

A prospective evaluation was performed at our ABA-verified burn center from June 1, 2023 to August 13, 2024. Patients were enrolled if burns < 20% total body surface area were autografted and antimicrobial therapy for burn cellulitis at the surgical site was continued post-operatively. Post-operative dressings were removed on post-operative day (POD) 3 rather than our standard POD 5 to evaluate wounds and determine if clinical signs of infection were present. Clinical evidence of infection was defined by erythema, purulence, edema, and wet-appearing grafts. A wound culture was obtained at takedown regardless of wound appearance. Antibiotics were continued if clinical signs of infection were noted and discontinued if signs of infection were absent.

**Results:**

There were 17 patients included, and 14 had antibiotics stopped on day of POTD. Of the 14 patients who had antibiotics stopped, none had autograft loss requiring repeat surgery. In the 3 cases in which antibiotics were continued on POTD, 1 patient had operating room tissue culture that grew Staphylococcus species and aerobic wound culture obtained on POD 3 that grew Klebsiella and Citrobacter species; 1 did not have operating room or aerobic cultures on POD 3; and 1 did not have operating room culture, but did have culture on POD 3 that grew moderate yeast. Of the 14 patients who had antibiotics stopped, 3 had growth on aerobic wound swab on POD 3, however none of these patients had autograft failure.

**Conclusions:**

The vast majority of patients had antibiotics successfully discontinued on the day of POTD without autograft loss. While few patients demonstrated microbial growth on post-operative takedown, it is unclear if this reflects infection rather than colonization.

**Applicability of Research to Practice:**

This study suggests that reducing the number of antibiotic days in burn patients through early removal of post-operative dressings and timely evaluation of the wound may be feasible without compromising patient outcomes. Further research is needed to refine protocols for antibiotic use in burn patients, focusing on understanding the implications of early antibiotic discontinuation.

**Funding for the Study:**

N/A

